# *Perrottetiataronensis* B.M.Barthol. & K.Armstr., sp. nov. (Dipentodontaceae), a new species from northwestern Yunnan Province, China and northern Kachin State, Myanmar and a re-examination of the Asian and Australasian taxa of *Perrottetia*

**DOI:** 10.3897/phytokeys.183.71505

**Published:** 2021-10-14

**Authors:** Bruce Bartholomew, Kate E. Armstrong, Rong Li, Peter W. Fritsch

**Affiliations:** 1 Dept. of Botany, California Academy of Sciences, 55 Music Concourse Dr., Golden Gate Park, San Francisco, California 94118-4503, USA California Academy of Sciences San Francisco United States of America; 2 Institute of Systematic Botany, New York Botanical Garden, 2900 Southern Blvd., Bronx, New York 10458-5126, USA Institute of Systematic Botany, New York Botanical Garden Bronx United States of America; 3 Key Laboratory for Plant Diversity and Biogeography of East Asia, Kunming Institute of Botany, Chinese Academy of Sciences, 132 Lanhei Rd., Heilongtan, Kunming 650201, Yunnan, China Kunming Institute of Botany, Chinese Academy of Sciences Kunming China; 4 Botanical Research Institute of Texas, 1700 University Drive, Fort Worth, Texas 76107-3400, USA Botanical Research Institute of Texas Fort Worth United States of America

**Keywords:** Huerteales, IUCN Red List, *Perrottetiaalpestris*, *Perrottetiaarisanensis*

## Abstract

*Perrottetiataronensis* from the Dulong Jiang valley in northwestern Yunnan Province, China and the Babulongtan mountain range in northern Kachin State, Myanmar is here described as a new species of the Dipentodontaceae. It is the third species of the genus to be recognized for China and the first to be reported for Myanmar. It is similar to *P.alpestriss.s.* but differs by characters of its leaf margins, inflorescences, and fruit. The three subspecies of *P.alpestris* recognized by Hou in “Flora Malesiana” are here recognized as three distinct species, i.e., *P.alpestris*, *P.moluccana*, and *P.philippinensis* on the basis of differences in diagnostic characters and distribution. The report in the “Flora of China” of the Taiwan species *P.arisanensis* from Yunnan is determined to be incorrect due to misidentification of two specimens at KUN.

## Introduction

*Perrottetia* Kunth is a genus of about twenty species occurring mainly in tropical America and tropical Asia. In addition, two species are endemic to the Hawaiian Islands (Lorence and Wagner 2019). The genus was formerly included in Celastraceae, but evidence from nuclear and plastid genes supports its placement in Dipentodontaceae (Zhang and Simmons 2006), now treated as a separate family in the order Huerteales (Chase et al. 2016). Dipentodontaceae has two genera, *Perrottetia* and the monospecific genus *Dipentodon* Dunn (Ma and Bartholomew 2008). In the Old World *Perrottetia* occurs from mainland Asia south to New Guinea and northeastern Australia, east to the southwest Pacific islands, and north to China and Myanmar. In the “Flora of China” two species were reported (Ma and Bartholomew 2008). One of these, *P.racemosa* (Oliv.) Loes., is widely distributed in southern and southwestern China (Ma and Bartholomew 2008), occurring in the provinces of Guangxi, Guizhou, SW Hubei, NW Hunan, Sichuan, and S–SE Yunnan as well as the Chongqing Municipality which was formerly the eastern part of Sichuan Province. *Perrottetiaracemosa* was first described as an *Ilex* L. by Oliver (1889) on the basis of collections made by A. Henry in Hubei Province, China, but was later transferred to *Perrottetia* by Loesner (1892). The other species in China, *P.arisanensis*Hayata (1915), was described from Taiwan Province. Recently, a third species has been found in China and Myanmar and is reported here in China from northwestern Yunnan Province in the Dulong Jiang valley of Gongshan Xian and in Myanmar from Kachin State in Putao District. This new species is similar to *P.alpestris* (Blume) Loesn. *s.s.* but differs by several characters given below in the discussion and the key to the Asian and Australasian species of *Perrottetia*. It is also disjunct from *P.alpestriss.s.* by over 2500 km.

The “Flora of China” treatment of *Perrottetia* reported that *P.arisanensis* not only occurs in Taiwan but also in central Yunnan in Eshan Xian 峨山县 and southeastern Yunnan in Xichou Xian 西畴县 (Ma and Bartholomew 2008). This Yunnan distribution is, however, incorrect as it was based on the misidentification of two specimens at KUN. The specimen from Eshan Xian (*Yuxi Team* 玉溪队 *89-256* collected 7 July 1989) has been redetermined to be a *Rhamnus* L., and the specimen from Xichou Xian (*Wu Quanan* 武全安 *7981* collected 7 May 1959) has been redetermined to be *P.racemosa*. With this correction, *P.arisanensis* is endemic to Taiwan.

## Taxonomy

### Perrottetia taronensis

Taxon classificationPlantaeStylommatophoraStreptaxidae

B.M.Barthol. & K.Armstr. sp. nov.

6152D7AA-6A69-5057-9C0C-EE4FF0B7312E

urn:lsid:ipni.org:names:77220797-1

Figures 1 , 2 , 3

#### Type.

China. Yunnan Province: Gongshan Xian 贡山县, Dulongjiang Xiang 独龙江乡, Maku Cun 马库村[Taron River], NW facing 30–60° slope, vicinity of Nangza (Pinyin: Laza) 腊咱, W side of the Dulong Jiang valley, ca. 1.3 direct km S of Maku and ca. 3.8 direct km NE of the Myanmar border, 1970 m, 27.6747°N, 98.3015°E, 18 August 2006, *Gaoligong Shan Biodiversity Survey 32394* (holotype: KUN! accession 0856752 barcode 1418097; isotypes: BRIT! barcode BRIT478072, CAS! accession 1090250 barcode 346898, E! barcode E01016879, GH! barcode 00288213).

#### Diagnosis.

*Perrottetiataronensis* is similar morphologically to *P.alpestriss.s.* (see Discussion) from which it differs by having much more compact and shorter inflorescences which are sparsely golden tan-tomentose rather than sparsely reddish brown-tomentose, a shorter stipe, leaf margins that are sharply serrate rather than bluntly serrate, and larger fruit when mature.

#### Description.

**Figure 1.**
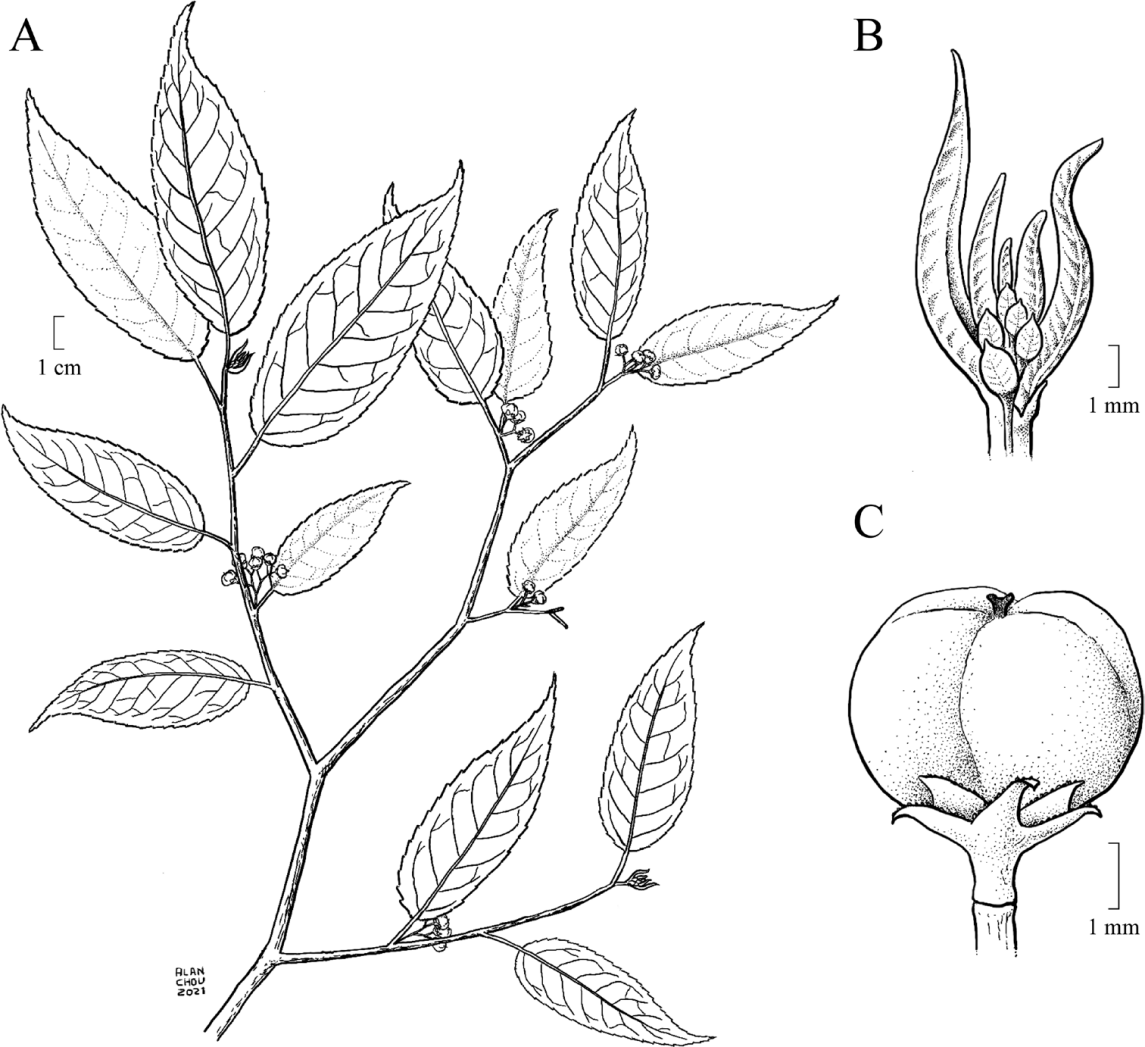
*Perrottetiataronensis***A** habit, branch with fruit **B** dactylose leaf bud with young leaves and stipules **C** fruit with style, mature berry, persistent sepals and petals, and stipe. Illustrated by Alan Chou.

**Figure 2.**
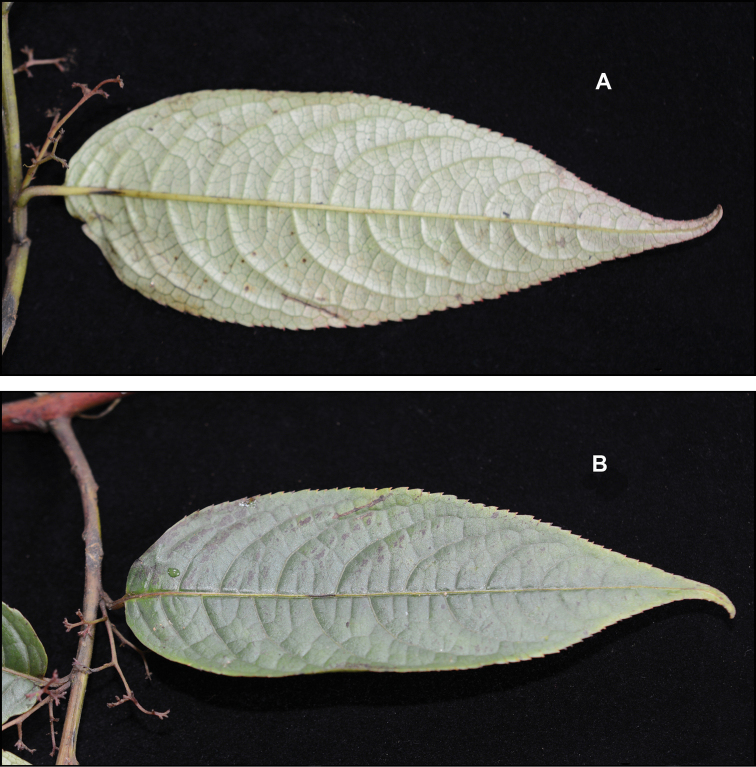
Leaves of *Perrottetiataronensis* showing also the old male inflorescences, from which all the flowers have dropped **A** abaxial surface **B** adaxial surface. Photos in the field of *Armstrong et al. 2983*.

**Figure 3.**
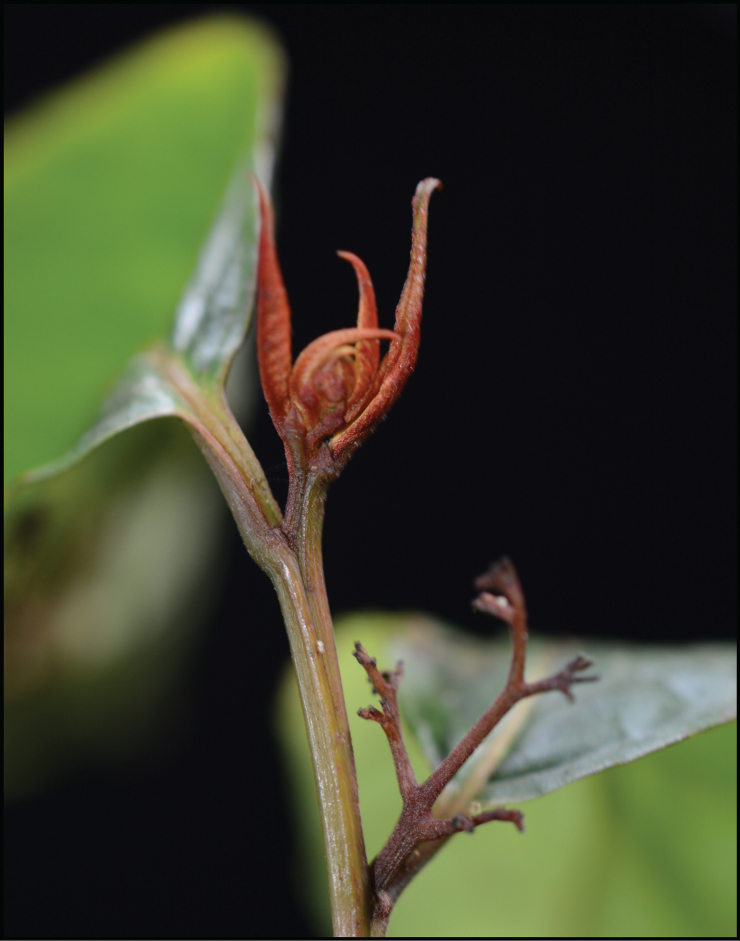
Young leaves of *Perrottetiataronensis* showing the distinctive dactylose shape and reddish brown indumentum and also an old male inflorescence, from which all the flowers have dropped. Photo in the field of *Armstrong et al. 2983*.

Shrubs or small trees 1.5–6.0 m tall, often sprawling, likely dioecious, deciduous. Stems reddish brown, pale brown-tomentose when young, glabrescent. Stipules reddish brown-tomentose, triangular, ca. 1.5 × 0.5 mm, often cauducous, apex long-acuminate. Petioles 0.5–1.0 cm. Leaves alternate, mostly glabrescent; leaf buds and young leaves dactylose (Figs 1B, 3), reddish brown-tomentose (Fig. 3), persistent as small naked buds over winter; mature leaf blades chartaceous, mostly glabrescent, sometimes with domatia abaxially in axils of main lateral veins, when fresh abaxially pale pinkish greenish (Fig. 2A) and adaxially dark green (Fig. 2B), when dry abaxially pale brownish green and adaxially dark green, narrowly ovate to elliptic, 10–15 × 3.0–7.5 cm, abaxially aveolate, sometimes sparsely tomentose on veins, midvein prominent, secondary and tertiary veins prominent, adaxially rugose, glabrous, midvein slightly prominent, secondary and tertiary veins slightly depressed, base rounded to broadly cuneate and slightly asymmetric with margin narrowly recurrent onto petiole, margins sharply serrate with 0.5–0.8 mm forward-facing sharply pointed corniculate teeth 0.2–0.3 mm wide at their base, apex narrowly acuminate and often slightly curved. Inflorescences axillary, paniculate thyrses, 1–2 cm, ca. 25–40-flowered but much reduced in number in fruit, sparsely golden tan-tomentose, with ca. 1.2 × 0.5 mm narrowly triangular acuminate bracts, basal portion of inflorescences before the first branch 1–2 mm. Flowers and fruit with a basally articulate 0.5–0.6 mm stipe (Fig. 1C), pedicel 1.0–1.5 mm. Flowers 5-merous; sepals and petals only slightly differentiated, persistent in fruit (Fig. 1C). Calyx tube broadly obconical, ca. 0.5 × 1 mm, lobes narrowly triangular, 1.0–1.2 × 0.5–0.6 mm, basally overlapping corolla lobes, margins minutely denticulate, apex acuminate and often distally reflexed. Corolla lobes broadly triangular, 1.0–1.2 × 0.5–0.7 mm, margins minutely denticulate, apices broadly acute. Stamens 5, at the edge of the floral disc and alternating with corolla lobes. (Only one male plant of *Perrottetiataronensis* has been seen. It has two remnant undeveloped flower buds with all other flowers already fallen. In *Perrottetia* the filaments elongate after the male flowers open, so the filament characters in the observed unopened flower buds of *P.taronensis* are likely not typical of what they would be during anthesis.) Anthers globular, ca. 0.3 × 0.4 mm. Ovary superior, turbinate, ca. 1.2 × 1.0 mm. Fruit a berry, young fruit green but starting to turn red by July and turning purple and becoming fleshy when mature by August, ca. 5 mm in diam. when mature, usually 4-seeded although occasionally with only 2 or 3 seeds developing, apex emarginate; style ca. 0.2 mm, often deciduous, apically 2-parted. Seeds brown, 1.0–1.5 mm in diam., surface with numerous shallow vertical rugose ridges when dry.

#### Etymology.

**Figure 4.**
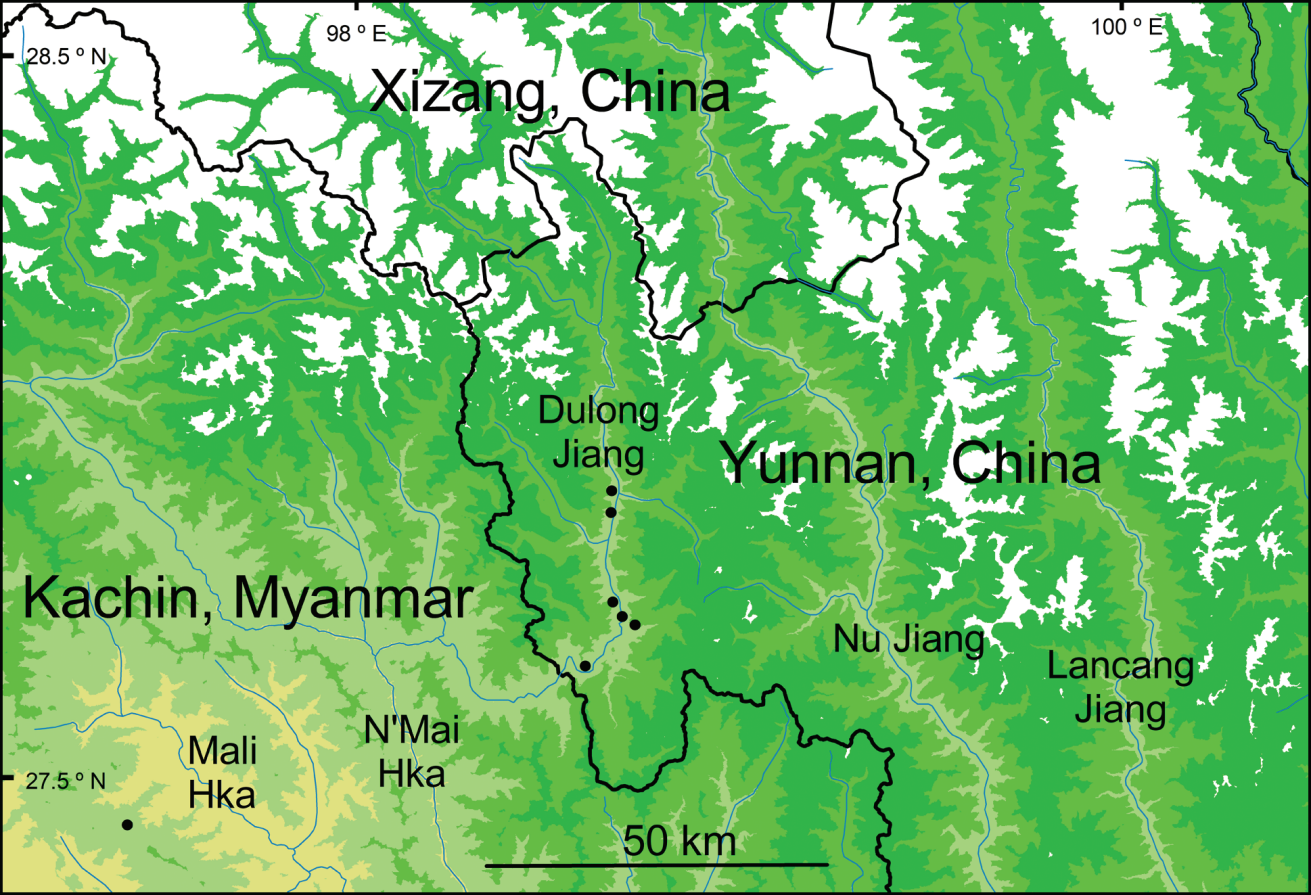
Distribution showing the known locations of *Perrottetiataronensis* in China and Myanmar. The map uses Shuttle Radar Topography Mission (SRTM) 90 m raster elevation data from Jarvis A, Reuter HI, Nelson E, and Guevara E, 2008, Hole-filled seamless SRTM data V4, International Centre for Tropical Agriculture (CIAT) available from https://srtm.csi,cgiar.org (Reuter et al. 2007). The elevation intervals for the region shown in this map have been set to 0–999 m, 1000–1999 m, 2000–2999 m, 3000–3999 m, and 4000 m and above. All seven collections are from within the 1000–1999 elevation interval shown on the map. Within China to the east and separated by the Gaoligong Shan range is the Nu Jiang (Salween River) and farther still to the east is the Lancang Jiang (Mekong River).

The specific epithet “taronensis” refers to the Taron River valley in Myanmar. In China this river is named the Dulong Jiang (Dulong River) (Fig. 4). The Taron River flows into the N’Mai Hka (N’Mai River) which joins the Mali Hka (Mali River) forming one of the main northern tributaries of the Ayeyarwady River (Irrawaddy River).

#### Habitat and distribution.

The seven collections of *Perrottetiataronensis* that have so far been made occur in the Ayeyarwady River drainage in both Yunnan Province, China and Kachin State, Myanmar at an elevation range of 1350–1970 m (Fig. 4). In China *P.taronensis* occurs on slopes in subtropical broadleaved evergreen forests, in disturbed secondary forests of *Alnusnepalensis* D. Don (an early successional tree species of secondary forests in this region), and among shrubs near river banks. In Myanmar, the habitat of the single collection of a male plant is classified within the Kachin Hills subtropical rainforest ecosystem, a closed-canopy humid lower montane forest type occurring between 700–1500 m (Armstrong et al. 2020; Murray et al. 2020). The six collections in China are from the valley of the Dulong Jiang which drains into the Taron River/N’Mai Hka in Myanmar, an upper tributary of the Ayeyarwady River. In Myanmar, the single collection is from the Babulongtan mountain range, in the drainage of the Nam Tisang/Mali Hka, which is in the adjacent river system to the west of the N’Mai Hka.

#### Proposed IUCN Conservation Status.

The proposed Conservation Status of *Perrottetiataronensis* is Endangered (EN), B1ab(i,ii,iii,v)+2ab(I,ii,iii,v), according to the IUCN Standards and Petitions Committee (2019). This status is based on the criteria of EOO 802.498 km^2^ and AOO 319.308 km^2^ calculated by using GEOCAT (Bachman et al. 2011) and due to the occurrence in a small area, a decline in the quality of its habitat, and the low number of mature individuals observed.

#### Paratype specimens of *Perrottetiataronensis* examined.

China. Yunnan Province: Gongshan Xian 贡山县, Dulongjiang Xiang 独龙江乡, Bapo Cun 巴坡村, in the vicinity of Bapo, on the E side of the Dulong Jiang, 1350 m, 27.74°N, 98.35°E (estimated coordinates), 19 November 1990, *Dulong Jiang Investigation Team* 独龙江考察队 *579* (CAS, KUN); Bapo Cun, W side of Gaoligong Shan, W of Gongshan, on the trail from Qiqi 其期 to Bapo in the Dulong Jiang valley, 1900 m, 27.7290°N, 98.3670°E, 17 July 2000, *Li Heng* 李恒 *12931 with Bruce Bartholomew, Philip Thomas, Peter Fritsch, Dao Zhiling* 刀志灵, *Wang Zhong-lang* 王仲朗 *& Li Rong* 李嵘 (CAS, E, MO); Bapo Cun, W side of the Gaoligong Shan, along Gamalai He (Gamalai River) 嘎莫赖河 and Dulong Valley on the trail from Xishaofang 西哨房 to Bapo, 1350 m, 27.7592°N, 98.3377°E, 18 July 2002, *Li Heng* 李恒 *15072 with Li Rong* 李嵘 *& Dao Zhiling* 刀志灵 (CAS, KUN); Kongdang Cun 孔当村, W side of Gaoligong Shan, along Dulongjiang Valley on the trail from Bapo to Dizhengdang 迪政当, 1550 m, 27.8773°N, 98.3355°E, 21 Jul 2002, *Li Heng* 李恒 *15156 with Li Rong* 李嵘 *& Dao Zhiling* 刀志灵 (CAS, KUN); Xianjiudang Cun 献九当村, W side of Gaoligong Shan along Dulong Jiang Valley on the trail from Kongdang to Dizhengdang, 1560 m, 27.9061°N, 98.3363°E, 23 July 2002, *Li Heng* 李恒*15230 with Li Rong* 李嵘 *& Dao Zhiling* 刀志灵 (CAS, KUN). Myanmar. Kachin State, Putao District, Naungmung Township, buffer zone of Hkakaborazi National Park, along trail between Maza and Namti, 1763 m, 27.4650°N, 97.6967°E, 18 June 2017, *Kate Armstrong 2983 with Thet Yu Nwe, Moe Myint Thu, San Naing Dee, Zaw Naing Tun, Hla Naing Htay & Pa Rang Gang Ken Sar* (CAS, E, NY, RAF).

## Discussion

As treated here *Perrottetia* is now known in Asia and Australasia as having six species of which three occur in China and one in Myanmar. The genus is herein recorded for the first time from Myanmar as it was not mentioned in either Merrill’s (1941) list of “Upper Burma Plants” or in Kress et al.’s (2003) country-wide checklist. On the basis of the single Myanmar collection being a male plant and all six Chinese collections made so far being female plants in fruit, it is likely that *P.taronensis* is dioecious. This condition is common in the genus, although additional field studies in the spring during flowering are needed for confirmation.

*Perrottetiataronensis* is most similar morphologically to *P.alpestriss.s.* (see below) which occurs in Peninsular Malaysia as well as in Indonesia on Sumatra and Java plus nearby small islands. It differs from *P.alpestriss.s.* by several characters enumerated in the diagnosis above and in the key to the Asian and Australasian species of *Perrottetia* below. Moreover, *P.taronensis* and *P.alpestris* exhibit a north-south disjunction of about 2500 km, with *Perrottetia* not recorded from intervening continental Southeast Asia (Hou 1962; Hou et al. 2010).

In addition to *Perrottetiaalpestriss.s.*, two other *Perrottetia* species (*P.moluccana* (Blume) Loes. and *P.philippinensis* (S. Vidal) Loes.) occur in Southeast Asia and Australasia. Although treated as species by Loesner (1892), they were treated respectively as P.alpestrissubsp.moluccana (Blume) Ding Hou and P.alpestrissubsp.philippinensis (S. Vidal) Ding Hou by Hou in the treatment in “Flora Malesiana” (1962). We follow Loesner in treating all three Southeast Asian and Australasian *Perrottetia* as separate species. Not only do they differ by several diagnostic characters as can be seen in the key to the Asian and Australasian species of *Perrottetia* below, but also *P.alpestriss.s.*, *P.moluccana*, and *P.philippinensis* are disjunct, as was pointed out by Hou (1962). *Perrottetiamoluccana* occurs on the island of New Guinea and the Cape York Peninsula of Queensland Australia, extending into some of the adjacent islands in the southwest Pacific. *Perrottetiaphilippinensis* occurs in Indonesia on the islands of Borneo and Sulawesi as well as in the Philippines and on some small adjacent islands. On the basis of diagnostic morphological characters, *P.alpestriss.s.*, *P.moluccana*, *P.philippinensis*, and *P.taronensis* are more similar to each other than this group of four species is to either of the other two Asian species, *P.arisanensis* and *P.racemosa*, and these similarities and differences are expressed in the key below. It is hoped that future molecular studies will confirm our interpretation based on diagnostic morphological characters.

We only know of seven collections of *Perrottetiataronensis* all of which were previously misidentified as *Celastrus* L. (Celastraceae), *Gaultheria* L. (Ericaceae), *Ilex* (Aquifoliaceae), *Maesa* Forssk. (Primulaceae), or *Rhamnus* (Rhamnaceae), so it is possible that other specimens are already in herbaria and may come to light, although a search in likely families and genera at KUN as well in the online Chinese Virtual Herbarium (https://www.cvh.ac.cn/) has so far not found additional specimens of this species.

### Key to the Asian and Australasian species of *Perrottetia*

**Table:** 

1	Inflorescences thyrses in a raceme; fruit apex rounded	***P.racemosa***
–	Inflorescences thyrses in a panicle; fruit apex emarginate	**2**
2	Corolla and calyx lobes ligulate, undifferentiated, not overlapping, ca. 1.0 × 0.2–0.4 mm, glabrous, margins entire; style in fruit 1.0–1.4 mm, usually persistent; inflorescences glabrous	***P.arisanensis***
–	Corolla and calyx lobes triangular, slightly differentiated, calyx lobes ca. 1.0 × 0.5–0.6 mm and overlapping corolla lobes, corolla lobes ca. 0.5–1.2 × 0.3–1 mm, margins of both calyx and corolla lobes minutely denticulate; style in fruit ca. 0.2 mm, often deciduous; inflorescences sparsely tomentose	**3**
3	Leaf blades thinly coriaceous, margins entire; stems conspicuously lenticellate	***P.moluccana***
–	Leaf blades chartaceous, margins serrate; stems not conspicuously lenticellate	**4**
4	Flowers 4-merous; inflorescences 5–10 cm with basal portion before first branching 2–3(–4) cm; serrations of leaf blade margins blunt with serrations 0.3–0.4 mm wide at their base; stipules apically acute	***P.philippinensis***
–	Flowers 5-merous; inflorescences 1–4 cm with basal portion before first branching 0.1–1.0 cm; serrations of leaf blade margins blunt or sharp with serrations 0.2–0.3 mm wide at their base; stipules apically acuminate	**5**
5	Inflorescences 2–4 cm, sparsely reddish brown-tomentose, basal portion before first branching 0.5–1.0 cm; stipe ca. 1.5 mm; leaf blade serrations apically blunt; fruit when mature and fleshy 3–3.5 mm in diam.	***P.alpestris***
–	Inflorescences 1–2 cm, sparsely golden tan-tomentose, basal portion before first branching 0.1–0.2 cm; stipe 0.5–0.6 mm; leaf blade serrations apically sharply pointed; fruit when mature and fleshy ca. 5 mm in diam.	***P.taronensis***

## Supplementary Material

XML Treatment for Perrottetia taronensis

## References

[B1] Armstrong KE Tizard R Grantham H (2020) Kachin Hills subtropical rainforest. In: Murray NJ Keith DA Tizard R Duncan A Htut WT Hlaing N Oo NA Ya KZ Grantham H (Eds) Threatened ecosystems of Myanmar: An IUCN Red List of Ecosystems Assessment.Version 1.0. Wildlife Conservation Society, 89–93.

[B2] Bachman S Moat J Hill AW de la Torre J Scott B (2011) Supporting Red List threat assessments with GeoCAT: Geospacial conservation assessment tool. In: Smith V Penev L (Eds) E-infrastructures for data published in biodiversity science.ZooKeys 150: 117–126. 10.3897/zookeys.150.2109 [accessed 12 July 2021] PMC3234434 22207809

[B3] Chase MW Christenhusz MJM Fay MF Byng JW Judd WS Soltis DE Mabberley DJ Sennikov AN Soltis PS Stevens PF (2016) An update of the Angiosperm Phylogeny Group classification of the orders and families of flowering plants: APG IV. Botanical Journal of the Linnean Society 181(1): 1–20. 10.1111/boj.12385

[B4] Hayata B (1915) Contributions to the flora of Formosa III. Icones Plantarum Formosanarum nec non et Contributiones ad Floram Formosanam 5: 1–349.

[B5] Hou D (1962) 12. *Perrottetia*. In: Van Steenis CGGJ (Ed.) Flora Malesiana ser.I, Vol. 6(2). Wolters-Noordhoff, Groningen, 288–291.

[B6] Hou D Savinov IA van Welzen PC (2010) Celastraceae. In: Santisuk T Larsen K (Eds) Flora of Thailand 10(2).The Forest Herbarium, Department of National Parks, Wildlife and Plant Conservation, Bangkok, 141–198.

[B7] IUCN Standards and Petitions Committee (2019) Guidelines for using the IUCN Red List Categories and Criteria. Version 14. Prepared by the Standards and Petitions Committee. http://www.iucnredlist.org/documents/RedListGuidelines.pdf [accessed 12 July 2021]

[B8] Kress WJ DeFilipps RA Farr E Kyi DYY (2003) A checklist of the trees, shrubs, herbs, and climbers of Myanmar. Contributions from the United States National Herbarium 45: 1–590.

[B9] Loesner T (1892) Celastraceae In: Engler HGA Prantl KA (Eds) Die Natürlichen Pflanzenfamilien 3(5).Verlag von Wilhelm Engelmann, Leipzig, 189–221.

[B10] Lorence DH Wagner WL (2019) *Perrottetiawichmaniorum* (Dipentodontaceae), a new species from Kaua’i, Hawaiian Islands. PhytoKeys 115: 93–103. 10.3897/phytokeys.115.30657 PMC6365489 30740022

[B11] Ma JS Bartholomew B (2008) Dipentodontaceae In: Raven PH Wu CY Hong DY (Eds) Flora of China Vol.11. Sciences Press, Beijing, and Missouri Botanical Garden Press, St. Louis, 494–495. http://flora.huh.harvard.edu/china/mss/volume11/Dipentodontaceae.pdf

[B12] Merrill ED (1941) The Upper Burma plants collected by Captain F. Kingdon Ward on the Vernay-Cutting expedition, 1938–39. Brittonia 4: 20–188. 10.2307/2804985

[B13] Murray NJ Keith DA Duncan A Tizard R Ferrer-Paris JR Worthington TA Armstrong K Nyan H Win TH Aung HO Kyaw ZY Grantham H (2020) Myanmar’s terrestrial ecosystems: Status, threats and conservation opportunities. Biological Conservation 252: 108834. 10.1016/j.biocon.2020.108834

[B14] Oliver D (1889) *Ilexracemosa* Oliver. Hooker’s Icones Plantarum 19(3): [sub pl.] 1863.

[B15] Reuter HI Nelson A Jarvis A (2007) An evaluation of void-filling interpolation methods for SRTM data. International Journal of Geographical Information Science 21(9): 983–1008. 10.1080/13658810601169899

[B16] Zhang LB Simmons MP (2006) Phylogeny and delimitation of the Celastrales inferred from nuclear and plastid genes. Systematic Botany 31(1): 122–137. 10.1600/036364406775971778

